# Supplementary material for: Distinct protocerebral neuropils associated with attractive and aversive female-produced odorants in the male moth brain
Source: eLife. 2021 May 14;10:e65683. doi: 10.7554/eLife.65683 (PMC8154038; doi:10.7554/eLife.65683)
Supplement: Figure 3—source data 1. [file elife-65683-fig3-data1.docx]

| **Figure 3 – Supplementary Table 1** Overview of individual projection neuron morphologies | | | | | |
| --- | --- | --- | --- | --- | --- |
| **Type** | **ID** | ***N*** | **Soma** | **AL innervations** | **Protocerebral innervations** |
| **mALT** |  |  |  |  |  |
| Pm_a | Cu-mALT1 | 1 | MC | Cu | Ca, VLP, SLP, SIP |
| Pm_a | Cu-mALT2 | 1 | MC | Cu | Ca, VLP, SLP, SIP |
| Pm_a | Cu-mALT3 | 1 | MC | Cu | Ca, VLP, SLP § |
| Pm_a | Cu-mALT4 | 2 | MC | Cu | Ca, VLP, SLP § |
| Pm_a | Cu-mALT5 | 1 | MC | Cu | Ca, VLP, SLP |
| Pm_a | Cu-mALT6 | 1 | MC | Cu | Ca, VLP, SLP § |
| Pm_a | Cu-mALT7 | 1 | MC | Cu | Ca, VLP, SLP |
| Pm_a | Cu-mALT8 | 1 | MC | Cu | Ca, VLP, SLP, SIP |
| Pm_a | Cu-mALT9 | 1 | MC | Cu | Ca, VLP, SLP, SIP |
| Pm_a | Cu-mALTa | 1 | MC | Cu | Ca, VLP, SLP § |
| Pm_a | Cu-mALTb | 1 | MC | Cu | Ca, VLP, SLP, SIP |
| Pm_a | Cu-mALTc | 1 | MC | Cu | Ca, VLP, SLP |
| Pm_a | Cu-mALTd | 1 | MC | Cu | Ca, VLP, SLP |
| Pm_a | Cu-mALTe | 1 | MC | Cu | Ca, VLP, SLP § |
| Pm_a | Cu-mALTf | 1 | MC | Cu | Ca, VLP, SLP, SIP |
| Pm_a | Cu-mALTg | 1 | MC | Cu | Ca, VLP, SLP, SIP |
| Pm_a | dma-mALT1 | 1 | MC | dm-a | Ca, LH, VLP |
| Pm_a | dma-mALT2 | 3 | MC | dm-a | Ca, LH, VLP |
| Pm_a | dma-mALTa | 1 | MC | dm-a | Ca, LH, VLP |
| Pm_a | dma-mALTb | 1 | MC | dm-a | Ca, LH, VLP |
| Pm_a | dmaPCx-mALTa | 1 | MC | dm-a, PCx Gs | Ca, LH, VLP |
| Pm_a | dmp-mALTa | 1 | MC | dm-p | Ca, LH, VLP |
| Pm_a | dmp-mALTb | 1 | MC | dm-p | Ca, LH, VLP |
| Pm_a | dmp-mALTc | 1 | MC | dm-p | Ca, LH, VLP |
| Pm_a | dmp-mALTd | 1 | MC | dm-p | Ca, LH, VLP |
| Pm_a | MGC^dmp^-mALT1 | 1 | MC | Cu, dm-p, dm-a | Ca, LH, VLP |
| Pm_a | MGC-mALTa | 1 | MC | Cu, dm-p, dm-a | Ca, LH, VLP |
| Pm_a | MGC-mALTb | 1 | MC | Cu, dm-p, dm-a | Ca, LH, VLP |
| Pm_a | MGC^dma^-mALTc | 1 | MC | Cu, dm-a, dm-p | Ca, VLP |
| **mlALT** |  |  |  |  |  |
| Pml_a | MGC-mlALT1 | 1 | LC | Cu, dm-a, dm-p | VLP |
| Pml_a | MGC-mlALT2 | 1 | LC | Cu, dm-a, dm-p | VLP |
| Pml_b | MGC^Cu^-mlALTa | 1 | LC | Cu, dm-p, dm-a, PCx G | VLP, SLP, SIP |

Ca, calyces; CB, central body; LC, lateral cell body cluster; LH, lateral horn; MC, medial cell body cluster; SIP, superior intermediate protocerebrum; SLP, superior lateral protocerebrum; VLP, ventrolateral protocerebrum.
