# Supplementary material for: Distinct protocerebral neuropils associated with attractive and aversive female-produced odorants in the male moth brain
Source: eLife. 2021 May 14;10:e65683. doi: 10.7554/eLife.65683 (PMC8154038; doi:10.7554/eLife.65683)
Supplement: Transparent reporting form [file elife-65683-transrepform.pdf]

eLIFE
1st Floor
24 Hills Road
Cambridge CB2 1JP, UK
P 01223 855340
W elifesciences.org
T @elife

eLife's transparent reporting form

We encourage authors to provide detailed information *within their submission* to facilitate the interpretation and replication of experiments. Authors can upload supporting documentation to indicate the use of appropriate reporting guidelines for health-related research (see EQUATOR Network), life science research (see the BioSharing Information Resource), or the ARRIVE guidelines for reporting work involving animal research. Where applicable, authors should refer to any relevant reporting standards documents in this form.

If you have any questions, please consult our Journal Policies and/or contact us: editorial@elifesciences.org.

Sample-size estimation

- You should state whether an appropriate sample size was computed when the study was being designed
- You should state the statistical method of sample size computation and any required assumptions
- If no explicit power analysis was used, you should describe how you decided what sample (replicate) size (number) to use

Please outline where this information can be found within the submission (e.g., sections or figure legends), or explain why this information doesn't apply to your submission:

In this study, no power analysis nor specific sample sizes justification was performed. With regards to the calcium imaging data, the sample size of eight was designed, as similar sample sizes (n: 6 to 9) has previously proven sufficient to demonstrate general response tendencies across the glomerular units (Sachse & Galizia, 2002; Wu, Xu, Hou, Huang, Dong, & Wang, 2015). As for sharp intracellular recordings, sample sizes are completely dependent on time and success-rate restrictions. We gathered as much data as possible within a three-year window.

Replicates

- You should report how often each experiment was performed
- You should include a definition of biological versus technical replication
- The data obtained should be provided and sufficient information should be provided to indicate the number of independent biological and/or technical replicates
- If you encountered any outliers, you should describe how these were handled
- Criteria for exclusion/inclusion of data should be clearly stated
- High-throughput sequence data should be uploaded before submission, with a private link for reviewers provided (these are available from both GEO and ArrayExpress)

Please outline where this information can be found within the submission (e.g., sections or figure legends), or explain why this information doesn't apply to your submission:

eLife Sciences Publications, Ltd is a limited liability non-profit non-stock corporation incorporated in the State of Delaware, USA, with company number 5030732, and is registered in the UK with company number FC030576 and branch number BR015634 at the address 1st Floor, 24 Hills Road, Cambridge CB2 1JP | August 2014

-We collected data whenever the insect materials were available.

-With respect to the intracellular recordings, each stimulus was presented in multiple trials, and in several distinct animals. As such, both technical and biological replication was considered, respectively. Details see Method and material. Comparisons across trials for the low-concentration protocol is available in the supplementary materials, while between subject variation is addressed in the main results. For the calcium imaging experiments, an example of across-trial variation is presented in Fig. 1C, while between-subjects variability was considered in Fig. 1D.

-Examples of all reported data is presented in the main article, while more detailed summaries and individual neuron data can be found in the supplementary materials.

-None of the odor responses were considered to be outliers. For the calcium imaging data, the staining procedure was previously described (e.g. Ian, Kirkerud, Galizia and Berg,2017; Chu, Heinze, Ian, & Berg, 2020), and the post scanning confirmed that the responses was sampled from the target neuron population. Intracellularly recorded responses would not be appropriate to exclude, as the electrophysiological data was evidently linked with specific labelled morphologies.

-For the intracellular data, we solely included medial- and mediolateral-tract projection neurons arborizing exclusively/primarily in the MGC. All labelled neurons that did not display such characteristics were excluded, irrespective of physiological responses to odor stimulation.

-No sequence data were utilized in this study.

Statistical reporting

- Statistical analysis methods should be described and justified
- Raw data should be presented in figures whenever informative to do so (typically when N per group is less than 10)
- For each experiment, you should identify the statistical tests used, exact values of N, definitions of center, methods of multiple test correction, and dispersion and precision measures (e.g., mean, median, SD, SEM, confidence intervals; and, for the major substantive results, a measure of effect size (e.g., Pearson's r, Cohen's d)
- Report exact p-values wherever possible alongside the summary statistics and 95% confidence intervals. These should be reported for all key questions and not only when the p-value is less than 0.05.

Please outline where this information can be found within the submission (e.g., sections or figure legends), or explain why this information doesn't apply to your submission:

Test statistics, p-values, as well as parameters related to central tendencies and dispersion, were presented where suitable (i.e. in the main results and/or in figure legends).

(For large datasets, or papers with a very large number of statistical tests, you may upload a single table file with tests, Ns, etc., with reference to sections in the manuscript.)

Group allocation

- Indicate how samples were allocated into experimental groups (in the case of clinical studies, please specify allocation to treatment method); if randomization was used, please also state if restricted randomization was applied
- Indicate if masking was used during group allocation, data collection and/or data analysis

Please outline where this information can be found within the submission (e.g., sections or figure legends), or explain why this information doesn't apply to your submission:

Not relevant.

Additional data files ("source data")

- We encourage you to upload relevant additional data files, such as numerical data that are represented as a graph in a figure, or as a summary table
- Where provided, these should be in the most useful format, and they can be uploaded as "Source data" files linked to a main figure or table
- Include model definition files including the full list of parameters used
- Include code used for data analysis (e.g., R, MatLab)
- Avoid stating that data files are "available upon request"

Please indicate the figures or tables for which source data files have been provided:

-Spike trains from all reported intracellular recordings are either displayed in the main results, or in the supplementary materials. The calcium imaging data is only reported as graphical formats, the detailed analysis of the raw data was described in the methods and materials section. An example of the raw data was given in Fig. 1b.
-All calculations performed in the data analysis are described in the methods and materials section.
